# Climate-proofing a malaria eradication strategy

**DOI:** 10.1186/s12936-021-03718-x

**Published:** 2021-04-17

**Authors:** Hannah Nissan, Israel Ukawuba, Madeleine Thomson

**Affiliations:** 1 Grantham Research Institute for Climate Change and the Environment, London School of Economics and Political Science, London, UK; 2 International Research Institute for Climate and Society, Columbia University, Palisades, NY USA; 3 Mailman School for Public Health, Columbia University, New York, NY USA; 4 Columbia University, New York, NY USA

**Keywords:** Malaria eradication, Disease modelling, Disease programming, Climate change, Climate variability, Policy, Monitoring and evaluation

## Abstract

Two recent initiatives, the World Health Organization (WHO) Strategic Advisory Group on Malaria Eradication and the Lancet Commission on Malaria Eradication, have assessed the feasibility of achieving global malaria eradication and proposed strategies to achieve it. Both reports rely on a climate-driven model of malaria transmission to conclude that long-term trends in climate will assist eradication efforts overall and, consequently, neither prioritize strategies to manage the effects of climate variability and change on malaria programming. This review discusses the pathways via which climate affects malaria and reviews the suitability of climate-driven models of malaria transmission to inform long-term strategies such as an eradication programme. Climate can influence malaria directly, through transmission dynamics, or indirectly, through myriad pathways including the many socioeconomic factors that underpin malaria risk. These indirect effects are largely unpredictable and so are not included in climate-driven disease models. Such models have been effective at predicting transmission from weeks to months ahead. However, due to several well-documented limitations, climate projections cannot accurately predict the medium- or long-term effects of climate change on malaria, especially on local scales. Long-term climate trends are shifting disease patterns, but climate shocks (extreme weather and climate events) and variability from sub-seasonal to decadal timeframes have a much greater influence than trends and are also more easily integrated into control programmes. In light of these conclusions, a pragmatic approach is proposed to assessing and managing the effects of climate variability and change on long-term malaria risk and on programmes to control, eliminate and ultimately eradicate the disease. A range of practical measures are proposed to climate-proof a malaria eradication strategy, which can be implemented today and will ensure that climate variability and change do not derail progress towards eradication.

## Background

A world free from malaria is a shared vision for the global health community. Fifty years ago, an attempt by the World Health Organization (WHO) to rid the world of malaria once and for all ended in failure and loss of morale, despite advances that led to elimination in several countries. Now, the global malaria community is again considering whether and how to target complete malaria eradication. Two independent initiatives to assess the feasibility of eradication, the WHO Strategic Advisory Group on Malaria Eradication (SAG_ME_) and the Lancet Commission on Malaria Eradication, have recently published their recommendations [1, 2].

Although, unlike the Lancet Commission, the SAG_ME_ concluded that setting a target date for eradication is premature, both reports set out an agenda for achieving eradication in the near future. Underpinning their recommendations is an assessment of how long-term trends in several drivers of malaria risk, including climate change, might benefit or hinder a push for eradication. Both reports rely on a climate-driven model of malaria transmission to conclude that long-term trends in climate will assist eradication efforts overall and thus do not prioritize strategies to manage the effects of climate variability and change on malaria programming.

Malaria has always been understood as a climate-sensitive disease, with transmission historically associated with summer months in temperate zones and humid lowlands in tropical regions. Unusual weather conditions have often precipitated deadly epidemics. In recent years, a variety of statistical and dynamical transmission models of malaria transmission, driven by climate variables, have been used to predict likely changes in the geographic distribution of the disease in a warmer world, all else being equal [2, 3]. This simplistic approach to explaining the spatial and temporal dynamics of malaria at the global scale is contrasted with that of renowned malariologist, Hacket, who in 1937 said: “*everything about malaria is so moulded and altered by local conditions that it becomes a thousand different diseases and epidemiological puzzles. Like chess, it is played with a few pieces, but is capable of an infinite variety of situations*”. Hacket’s view suggests that little about malaria is predictable. These contrasting perspectives have been at the heart of a protracted debate on the likely importance of climate change to the future burden of malaria relative to other factors such as drug resistance [4–6].

**Fig. 1:**
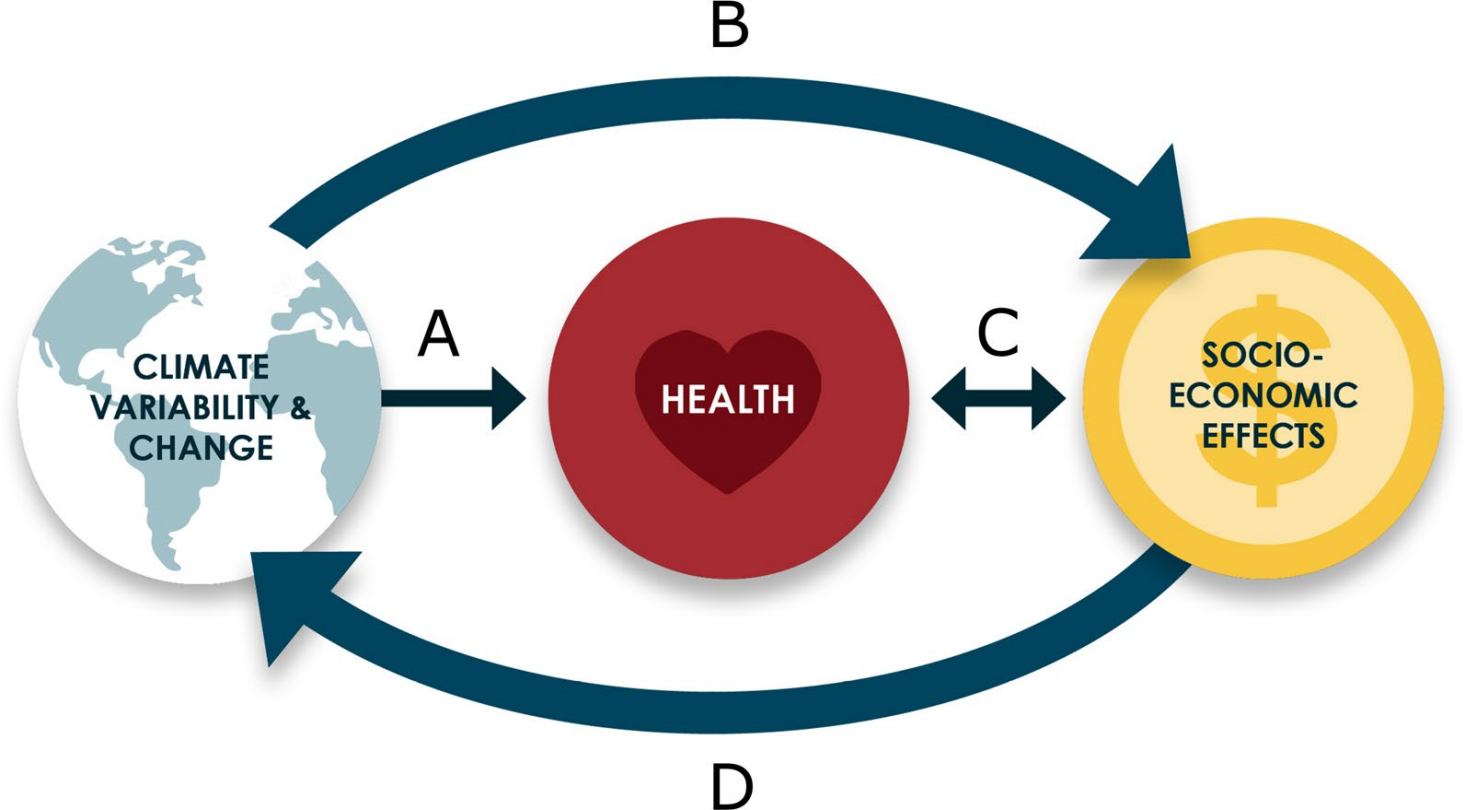
Direct and indirect interactions between climate and health. Conventional disease models consider only the direct effects of climate variability and change on health outcomes (A), but the climate also affects health outcomes indirectly, through its influence on the many socioeconomic factors that combine to determine health risks through a two-way process (as population health also influences socioeconomic outcomes) (B and C). The climate cannot be considered an exogenous part of this system: socioeconomic factors are driving climate change through greenhouse gas and aerosol emissions and land-surface changes (D) ( adapted from Thomson and Mason [82]; available from https://cipha.iri.columbia.edu/CIPHABOOK2019/Supplementary_Materials/)

To ensure that climate change does not derail malaria eradication activities going forward, it is important to understand which aspects of the relationship between climate and malaria transmission, morbidity and mortality are predictable at different spatial and temporal scales. Climate can influence malaria directly, through its effects on vector and parasite development and transmission dynamics, or indirectly, through myriad pathways including the many socioeconomic factors that combine to determine malaria risk (Fig. 1). Indeed, in 1969 the World Health Assembly acknowledged “*the part played by socio-economic, financial, administrative and operational factors, as also by the inadequacy of the basic health services, in the failures recorded during the implementation of the global malaria eradication programme*” [7]. We are, therefore, compelled to explore the full range of potential risks associated with the enormous environmental, social and economic impacts of climate change which may impact malaria eradication efforts through multiple possible pathways. Clearly, many of these pathways are unpredictable and thus cannot be included in models of malaria transmission, but failing to acknowledge the complexity of climate’s impact on malaria may render futile the malaria community’s efforts towards eradication. Predictive models are just one part of a pragmatic portfolio of activities, which must be complemented by other approaches including, for example, implementing strategies for managing risk under uncertainty and investments in better monitoring and surveillance.

Caution about an overreliance on predictive models is particularly important when planning for the long-term impacts of climate change on malaria risk. The most common approach to planning for climate change is to use long-term projections of rainfall and temperature from climate models to drive models of malaria transmission [2]. The outputs of these modelling experiments are then used to make plans for future programming under the scenarios predicted by the models. However, this approach has major problems which, as argued below and in previous publications [8, 9], could actually increase vulnerability to future climate variability and change. Fortunately, there are pragmatic alternatives to relying exclusively on long-term projections for practical planning. Complete malaria eradication is a long-term goal, and so any eradication strategy clearly must consider the potential evolution of risk drivers over the coming decades. Practically, however, an eradication strategy will be implemented through malaria control and elimination programmes in regions where malaria is prevalent, and programmes to prevent resurgence or spread of the disease in regions that are currently malaria free [1]. Thus, the primary tasks are to identify how climate may impact upon malaria control and elimination programmes and to identify regions where climate conditions may become suitable for resurgence or spread. Below, it is argued that these tasks do not predominantly require multi-decadal projections; rather, what is needed is an adaptive strategy that incorporates the changing effects of climate variability and change on malaria risk and control programmes. This strategy will require better monitoring of both climate and malaria, combined with skillful climate forecasts from weeks to a few years ahead. This pragmatic approach leads to actionable steps to achieving malaria eradication by focusing on the entry points within existing decision-making processes.

This paper, which has evolved from work commissioned by the SAG_ME_, proposes a new framework for incorporating climate into a malaria eradication strategy, which can be extended to long-term planning for a range of public health concerns. It asserts that the evidence supporting the claim that climate change will not pose a problem for eradication is weak and that climate should be considered carefully in the design of long-term malaria control, elimination and eradication programmes. Neglecting to integrate climate resilience into control efforts could ultimately result in failure to eradicate the disease, but also to delays in progress if windows of favourable climate conditions are not exploited. A key difference between the approach proposed here and that taken by the Lancet Commission is the emphasis herein on planning for an uncertain future, which draws on knowledge from the climate adaptation literature and experience from the humanitarian, engineering and infrastructure sectors—communities that have long grappled with the challenges of preparing for potential and uncertain climate impacts. The article also stresses the importance of managing the effects of climate variability on eradication efforts and cautions against an exclusive focus on long-term climate trends. The proposed approach offers practical, actionable steps that can be implemented today and which will ensure that climate variability and change do not derail progress towards malaria eradication.

## How climate influences malaria

### Climate variability and climate change: some basic concepts

The climate naturally varies on multiple timescales, from the daily weather and seasonal cycles to fluctuations occurring from year-to-year (inter-annual variability) and over longer cycles of 10–30 years (multi-decadal variability). This natural climate variability has been superimposed on a background of nonlinearly-increasing atmospheric greenhouse gas concentrations since around the start of the twentieth century. Rising greenhouse gas concentrations have resulted in detectable trends in average climate (particularly temperature), but also in changes in the timing of key seasons in some locations and in daily weather variability, including extreme weather and climate events like heat waves and droughts [10–12]. It is primarily through these changes in weather and seasonality, rather than through gradual, long-term trends, that climate change is likely to influence malaria risk. These impacts on malaria could occur both directly, as optimum climate ranges and critical thresholds for vector and parasite development are crossed, and indirectly, as society grapples with the disruptive effects of changes in weather patterns and seasonal cycles.

**Fig. 2:**
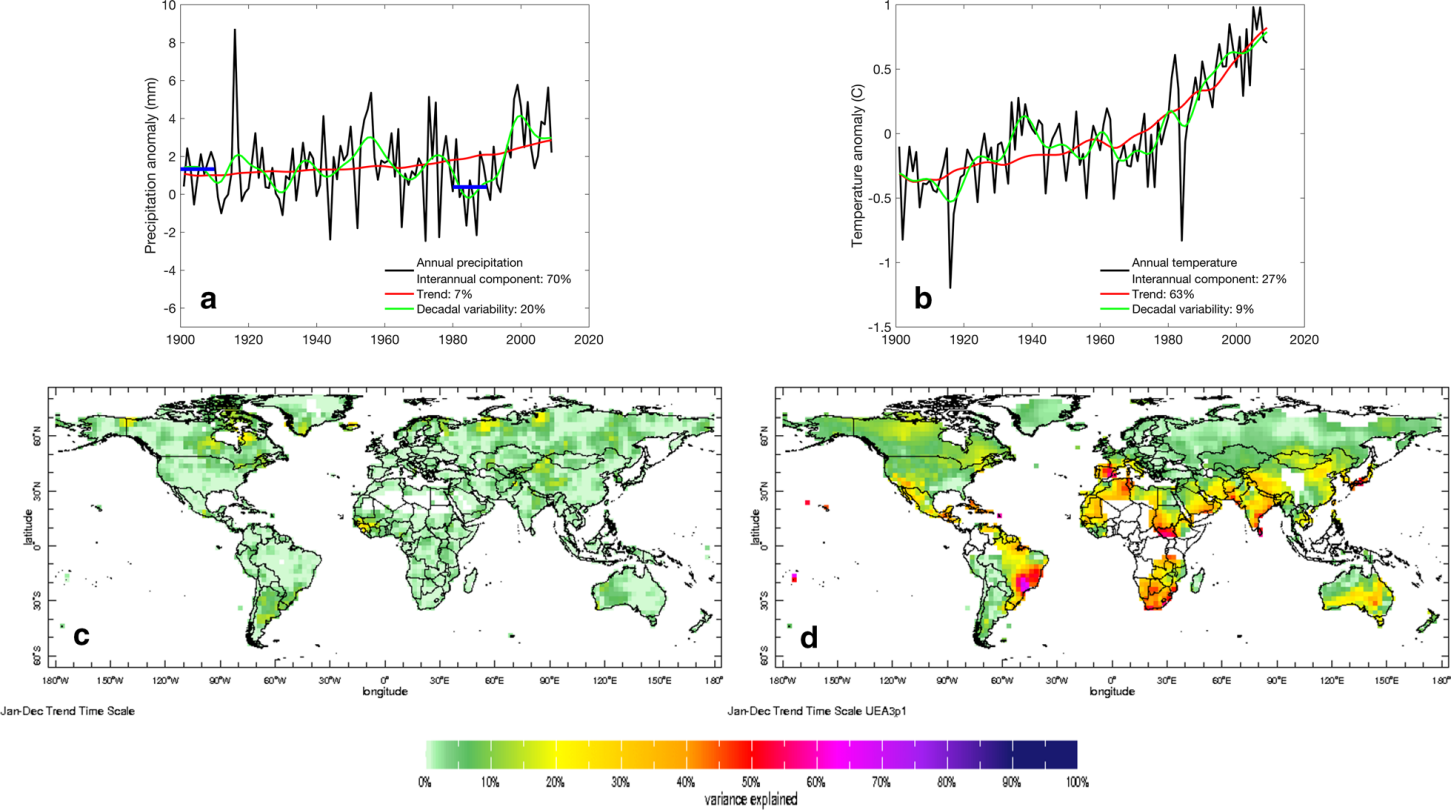
Timescales of variability for global average annual precipitation (**a**, mm) and temperature (**b**, °C) anomalies. Raw annual averages are shown in black, fitted decadal cycles in green and the long-term trend in red. In (**a**), the horizonal blue lines show the 10-year average precipitation anomalies from 1900–1910 and 1980–1990 (see text). The portion of total variance (%) in precipitation (**c**) and temperature (**d**) explained by the long-term trend is shown spatially in the bottom two panels. See Greene et al. [83] or http://iridl.ldeo.columbia.edu/maproom/Global/Time_Scales/index.html for the methodology and data

Two observations are immediately clear from the different timescales of rainfall and temperature variations shown in Fig. 2a and b. The first observation is that the global temperature trend is much more clearly discernible above the background of year to year and decadal variability than is the trend in precipitation (time series panels). This difference can be seen at the global scale, but it is also true at smaller scales, where the percent of total variance in annual rainfall amount that can be attributed to the trend over the last century is less than 10% almost everywhere (map panels). The second observation is that, for both rainfall and temperature, interannual and decadal variability together account for a much greater proportion of the fluctuations in local climate than the trend in almost all regions of the world. Notable exceptions for temperature can be seen in southeast Brazil, southern Africa, South Sudan, Spain and certain pockets in South Asia. What is the significance of these observations? Given the non-linear dependence of malaria transmission on changes in temperature and rainfall, climate variability has much more important implications for efforts to control and eliminate malaria than gradual long-term trends, particularly for rainfall. Understanding how climate variations across timescales may affect disease management strategy is thus an essential starting point to addressing if and how potential future changes in the climate should be considered.

Because the climate fluctuates so much, great care has to be taken when determining whether observed or projected changes in climate are symptomatic of a long-term climate change signal, or in fact just part of natural year to year or decadal climate variability. Confusing these conclusions would result in very different recommendations for how to prepare for the impacts of climate change on malaria. For this reason, the World Meteorological Organization recommends that trends are only calculated between windows of at least 30 years in duration. Otherwise, the computed trend is likely to be biased by interannual or decadal fluctuations. For example, globally, precipitation has increased slightly since the start of the twentieth century (Fig. 2a). However, if the change in rainfall is calculated between the period 1900–1910 and 1980–1990, one could easily conclude that in fact the world has been drying overall (Fig. 2a) (it has in fact been drying in places, though globally there has been a slight increase). This interpretive error is often made when projecting climate change into the future and could severely distort the conclusions of modelling studies which use climate projections to drive malaria (or other) health impact models. If future climate scenarios are calculated for individual years (as in the recent Lancet commission on malaria eradication), it would be quite possible to conclude that rainfall will increase in a particular place, when in fact the overall trend could be drying (for example).

### Direct effects of climate on malaria

#### Temperature

Existing knowledge on the influence of temperature on malaria is derived from a mixture of modelling and laboratory studies on human and non-human malaria, as well as from field observations of epidemics in highland areas during unusually warm years [13], often associated with El Niño events [14]. Taken together, these studies compile considerable evidence that, within a moderate temperature range, warmer conditions facilitate malaria transmission by influencing both the rate of parasite development and vector population dynamics. Below this range, a minimum temperature is required for mosquitos to survive long enough for parasite development to complete, and above it lethal temperature thresholds are reached which cause the adult population of mosquitos to drop sharply [3, 15–18]. The estimated shapes and optimum temperature-dependencies of the parasite and vector population are complex, do not correspond with each other and differ significantly among studies [17, 19, 20]. Accordingly, modelling work has demonstrated the complexity of the overall influence of temperature on transmission risk, producing substantially different estimates of optimum transmission temperatures according to assumptions made about the temperature dependencies and variability of each component [17, 20].

Different estimates of the transmission thresholds have important implications for understanding future malaria risk in a changing climate. For example, increasing night time temperatures have been observed, which could imply greater parasite and mosquito survival outside the usual malaria season in areas exhibiting seasonal transmission. Different temperature thresholds may also affect the future expansion and contraction of geographic malaria zones. If the maximum temperature thresholds for transmission are on the lower side of current estimates, more malaria control may be needed in temperate regions and less in hot areas, where conditions may be unsuitable for malaria transmission. Conversely, if the maximum transmission temperature is closer to upper estimates, rising temperatures could continue to exacerbate malaria risk in already hot regions.

Most modelling studies rely on outdoor average temperature data to simulate transmission risk. Mosquitoes can spend considerable time indoors and the association between indoor and outdoor conditions is generally understudied, particularly in developing countries [21]. There is also evidence from modelling work [21], supported by empirical evidence in Kenya [22], and laboratory experiments on rodent malaria [23] that diurnal temperature fluctuations (not captured by daily-averaged temperature data) moderate the temperature dependence of malaria transmission. A decrease in diurnal temperature range is one of the markers of climate change, as night-time temperatures have warmed faster than day-time temperatures since the 1950s [24, 25], though the change (including the direction of change) has been neither uniform across the globe nor constant with time [25]. The effects of such changes on transmission could be complex, especially if they occur close to transmission thresholds.

#### Rainfall

The primary pathway via which rainfall directly affects the malaria vector is by altering the number, quality and location of breeding sites for oviposition (egg-laying). The major African malaria vectors, *Anopheles gambiae *sensu stricto and *Anopheles arabiensis,* commonly inhabit small and sunlit temporary water pools. These pools can be as small as a hoof print as long as the pool does not dry out during the aquatic development phase of the mosquito. For example, in Botswana, variability in rainfall totals for the period December-February accounted for more than two-thirds of the interannual variability in standardized malaria incidence (January-May). However, the relationship of rainfall to malaria was nonlinear and extreme rainfall may have resulted in lower than expected malaria because floods eliminated breeding sites [26]. Moreover, the time between episodes of rainfall can result in phase amplification, allowing mosquito populations to grow rapidly, while longer spacing of rains can limit the growth rate of the mosquito population [27]. Under some circumstances drought can enhance vector breeding sites when water pools in dried-out dams and rivers. In Venuezuela malaria mortality has been shown to be more strongly related to drought in the year preceding outbreaks than to rainfall during epidemic years [28]. Droughts in the previous year may also depress population immunity making people more vulnerable to malaria once normal rains return [27].

#### Humidity

Adequate humidity is essential to mosquito survival as the insects are highly susceptible to desiccation. High humidity may precede heavy rainfall when temperatures are high, since moisture evaporating from the land surface in warm conditions is prevented from escaping by the arrival of clouds. Near the land surface, high relative humidity leads to an increase in mosquito survival, flight activity and host-seeking behaviour. These changes favour malaria transmission within an optimum relative humidity range of approximately 60–80% [29, 30].

#### Covariations between temperature, rainfall and humidity

Secondary pathways occur via the covariations between temperature, rainfall and humidity. Precipitation is usually associated with (at least temporary) reductions in near-surface temperatures. Therefore, while rainfall can enhance malaria transmission through its effect on breeding site abundance and humidity, this effect can be tempered by cooler conditions which may constrain malaria transmission or enhance it, depending on the proximity to critical temperature thresholds for transmission. Parham and Michael [31] argue that precipitation more strongly controls malaria endemicity than temperature through its influence on vector abundance, but that temperature has a greater influence on the rate of disease spread as long as sufficient rainfall exists to maintain a population of adult vectors. While El Niño events are generally associated with warming in the tropical belt they may also be associated with unusually high or low rainfall. The interaction of minimum and maximum temperature with rainfall may complicate the impact on malaria and care is needed to ascertain which variable(s) is(are) driving transmission at any particular locality [32]. Lag relationships exist between temperature, humidity, rainfall and their individual and combined effect on malaria transmission, increasing the difficulty of untangling relationships.

### Indirect effects of climate on malaria

Climate variability and change have many complex ramifications for socioeconomic systems and other processes relevant to disease control. Many of these indirect effects are understudied and unquantified, but an understanding of the potential pathways of influence and the manner in which they may materialize can inform the design of a successful long-term malaria eradication strategy by ensuring it is robust to a range of plausible, though uncertain, outcomes.

#### Impacts of climate variations across timescales

##### Extreme weather and climate events

Extreme weather and climate events include storm surge, tropical cyclones, heat and cold waves, heavy rainfall and flooding, which occur on timescales of hours up to a week or two, and drought, which results from a deficit in rainfall over a period of months. Changes in the frequency and severity of some types of extreme weather have already been detected, including heat waves and, in some regions and for some seasons, heavy rainfall [33]. An increase in frequency of heat extremes (and a decrease in cold extremes) is among the most robust predictions for future climate change [34], but future changes in extreme rainfall will vary substantially by region, with both increases and decreases occurring.

- *Logistics* One of the primary effects of extreme weather is disruption to the normal functioning of society. The practicalities of accessing diagnostics, drugs, vector control and vaccines become particularly challenging during periods of extreme weather. Roads and other transport lines may be out of service, complicating the transport of supplies and personnel, and compromising access to vulnerable populations, especially in remote rural areas.
- *Supply chains* As supply chains become increasingly monopolized, the continued provision of key commodities such as vaccines, drugs and ITNs becomes more vulnerable to disruption, both from climate shocks and non-climate factors like political unrest.
- *Vector control* Pooling water following periods of rainfall generally increases the availability of suitable breeding sites, but in some cases heavy rainfall has been observed to reduce vector abundance by flushing out and diluting existing sites. The nonlinearity of these mechanisms means that changes to the frequency, intensity and location of extreme rainfall episodes may have unpredictable effects on overall malaria transmission.
- *Behavioural factors* Excessive heat, particularly during the dry season, is a common barrier to the use of insecticide-treated nets (ITN) [35, 36] and may facilitate resurgence of malaria in some settings.
- *Vaccine supply* If a malaria vaccine (currently in pilot implementation) is deployed widely in the future, power outages and interruptions to normal storage and transport during extreme weather are likely to threaten the ‘cold chain’ [37, 38]. During heat waves, such breaks rapidly expose vaccines to high temperatures, particularly in tropical developing countries where power supply is often intermittent and may be dependent on hydropower.

##### Seasonality

Climate change is likely to cause changes in the malaria transmission season, for example through prolonged summer temperatures or changes in the timing of seasonal rainfall patterns. Shifts in seasonality may affect the impact of malaria control programmes and require a change in the timing of operations like Indoor Residual Spraying (IRS). Risk mapping may become less effective as changes in seasonality deviate from historical patterns used to inform baseline risk analyses.

##### Interannual climate variability

Variations in climate from year to year manifest as fluctuations in average temperature or total rainfall, but also as changes in the number or intensity of extreme events like droughts or heat waves, and in the timing of normal climatic events like the onset or cessation of dry and rainy seasons. Managing these variations is already a challenge for disease control programmes. In Botswana reduced prevalence of malaria during the drought of the early 1980s resulted in a lack of preparedness, high case numbers and then fatalities when normal rainfall returned in 1988 [26]. Interannual variability in climate can also affect the assessment of the impact of malaria interventions. For example, in Tanzania, the success of a scale-up of malaria control interventions was assessed relative to a baseline period that included the strongest El Niño event on record. The high rainfall amounts and unusually high temperatures associated with the El Niño event increased caseloads, thus predisposing the programme evaluation to success when the country subsequently experienced a series of droughts [39].

While changes in weather patterns and variability have already been observed, there is no evidence of increasing interannual variability over recent decades, although some modelling studies indicate that changes in interannual variability could occur in the future [34]. However, confidence is low in predictions of any change in interannual variability because climate models currently fail to capture both the variance and spatial pattern of El Niño Southern Oscillation, the dominant mode of interannual variability in the climate system [40].

##### Decadal climate shifts

In many regions of the world, climate exhibits slow cycles of increased/decreased rainfall or temperature over 10–20 year periods (Fig. 2). These cycles can easily be misconstrued as evidence of stable long-term trends from climate change; human (and political) memory tends not to extend much further than the past ten years and in many malaria regions climate records are too short to detect such slowly-changing patterns. Failure to recognize where recent climate patterns could constitute the rise or fall of decadal shifts rather than longer-term trends could lead to outbreaks if authorities are unprepared for a potential reversal in background climate suitability. The evaluation and thus the effectiveness of disease control programmes is compromised when this climate suitability is not considered. For example, malaria control efforts masked the influence of decadal temperature variability on malaria incidence in East Africa, which otherwise would have predisposed the region towards increased risk of outbreaks [41].

##### Long-term trends

In most places, lack of sufficiently-long data records on malaria incidence, vector control programmes and local climate have so far confounded efforts to discern the influence of long-term climate trends on malaria prevalence in the historical record. These efforts are further hindered by the nonlinearity both of the climate trends themselves (which makes delineating between decadal cycles and long-term trends challenging) and of the malaria response to changes in local climate. A recent study on the Ethiopian highlands (a rare example where reasonably long records of local climate and malaria data are available) confirmed that the elevations of minimum transmission thresholds have indeed been increasing, but also found considerable interannual and spatial variability in these thresholds, stressing the importance of high-quality data to monitor and understand variations in climate trends on the local scales at which malaria control programmes are implemented (Box 1) [42].

The magnitude of projected climate trends, as well as the timing at which relevant thresholds may be crossed, cannot be predicted accurately, particularly at local scales (see section “Climate-driven models of malaria transmission”). Nonetheless, considering how some long-term changes might *plausibly* influence malaria control strategies is important to ensure that programmes can adapt and respond to emerging risks:

- The expansion and contraction of climatically-suitable malaria zones as a result of long-term trends would be relevant for all components of a malaria control and eradication programme, potentially requiring a scale-up of new disease control efforts in previously unaffected areas and a review of resource allocation in newly eliminated areas. However, climate variability on all timescales means that these changes will not occur incrementally over time, and thus will need to be managed proactively from year to year.
- Coastal intrusion caused by slowly rising sea levels and brief storm surges may influence vector species in unknown ways, but is is thought most likely to favour transmission [43, 44].
- Upward trends in the concentration of carbon dioxide (CO_2_) in the atmosphere may have chemical effects on malaria risk, separate from their effects on altered climate and weather patterns. Elevated concentrations of carbon dioxide may be linked to delayed larvae development and increased mortality in woodland areas through an alteration in the chemical and nutritional quality of leaf litter [45].
- Trends in atmospheric CO_2_ may also affect the nutrition provided by staple crops, with consequences for malaria morbidity and mortality [46–48]. Rising CO_2_ concentrations also increase rates of plant growth [49]. Although this plant fertilisation effect provides a slow benefit to agricultural productivity, which may indirectly influence malaria control and eradication through indirect socioeconomic effects, it must be balanced against the complex and more immediate effects of changing weather patterns, seasonality and longer-term variability.

#### Cross-cutting climate issues affecting malaria control and eradication

##### Functioning health systems

Health systems are vulnerable to climate via two principle pathways: population access and government funding.

- *Population access* Extreme weather and climate events can disrupt operations, making it difficult for staff to reach work, for supplies to be transported and for people to access health care. Depressed household incomes following weather shocks, particularly in communities heavily dependent on agriculture, may prevent people from seeking medical attention when needed. As transmission dynamics evolve over time, in part influenced by climate, the constitution of vulnerable groups may change. For example, regions transitioning from endemic to epidemic will need to address a shift in susceptible age groups from children under 5 years to people of all ages [50]. Similarly, livelihoods, migration patterns and urbanisation are influenced by environmental change and bring new challenges to which health systems must respond to ensure that vulnerable populations can be identified and accessed.
- *Government funding* Currently, governments of endemic countries fund about 30% of malaria control activities [50]. Unless substantial progress towards adaptation is made, the economic impacts of climate change are likely to result in a squeeze on domestic funding for malaria control. Funding for health services may be particularly hard-pressed following extreme weather and climate shocks that cause a drop in Gross Domestic Product (GDP) and tax revenues.

##### Accurate risk mapping

Risk mapping to identify transmission hotspots (areas where climate and other factors make local transmission feasible) can be more efficient than disease surveillance in low or residual transmission areas and may help to target interventions appropriately [50]. As efforts to control malaria improve and the goal of eradication draws closer, residual transmission following earlier successful control programmes is likely to become a key focus. The changing character of weather and climate within seasons, throughout the year, and on longer timescales must be incorporated into risk maps in order to capture the changing features of transmission hotspots, but such efforts are hampered by a lack of quality data and insufficient case numbers in low transmission areas and seasons [51].

##### Effective insecticides

Overall, research on insecticide resistance suggests a complex response to temperature, with some studies reporting a decrease in effectiveness at higher temperatures and others an increase, depending on the type of insecticide and the range of temperatures tested [52–57]. Possible effects of changing climate and weather patterns on insecticide efficacy are therefore difficult to discern. Further research would be needed to identify important thresholds, and to investigate whether insecticides are affected differently by slow changes in ambient temperature (for example a warmer-than-usual season) or rapid heat and cold waves, factoring in any timelines in insecticide toxicity that may mediate the influence of temperature.

##### Climate action in other sectors

The malaria community should remain vigilant for the ways in which climate change adaptation and mitigation strategies may support or undermine malaria elimination and eradication strategies. For example, the use of DDT as a pesticide in agriculture to improve production led to benefits for public health, but may have contributed to insecticide resistance in Zimbabwe [56]. Water management practices evolve in response to climate stresses and market forces and can have unintended consequences for malaria transmission. In areas of unstable malaria transmission where local communities lack immunity, for instance in the African highlands and desert fringes, widespread irrigation to improve agricultural yields increases the number of mosquitoes and can lead to an increase in malaria incidence. However, in areas of stable transmission (most of sub-Saharan Africa) there is no evidence that irrigation impacts transmission rates. Despite an increase in mosquito density with irrigation, malaria risk may decline due to substitution with a mosquito of lower vectorial capacity which thrives in more irrigated land. In either case, improvements in the local economy as agricultural incomes grow and stabilize with irrigation generally lead to better ITN use and improved access to healthcare services compared with communities not using irrigation [58].

##### Climate impacts on food security

Food insecurity and nutrition are strongly affected by climate variability, with complex consequences for malaria risk and thus for appropriate control measures. Refeeding famine victims can increase malaria, depending on the amount and type of sustenance given, while in chronically malnourished (but not starving) populations protein energy malnutrition may increase malaria morbidity and mortality [59]. Nutritional supplementation to manage malaria risk could become a more important component of malaria control in the future in response to increased food insecurity and changes in nutrition related to climate change, but more evidence of the effects of supplementation is needed [59].

## Assessing the impacts of climate change on future malaria risk

### Climate-driven models of malaria transmission

In recent years mathematical models of malaria, of varying degrees of complexity, have been developed both to explain observed variations in cases and to predict future ones [60]. Unlike statistical models, which are based on empirical relationships between disease drivers and malaria transmission, dynamical models simulate the transmission dynamics and so are more suitable for exploring the effects of changes in disease drivers that have not been observed in the data record. However, dynamical models also rely on statistical relationships to approximate aspects of the model that are poorly understood so they must still be interpreted with caution. Non-linearities in the relationship of climate drivers to disease outcomes is an important reason for consulting dynamical transmission models, as long as their limitations are also considered. These mechanistic models can be driven either by climate data or forecasts on a range of timescales to explore changes in transmission resulting from observed or predicted climate variations [5, 61–65].

No model is ever perfect. A recent study used a climate-driven dynamical transmission model to quantify the relative significance of uncertainty in the climate data used to drive the model (in this case temperature observations), initial conditions (gametocyte carrier rates) and transmission model uncertainty in the simulation of observed clinical cases for Kericho in Kenya [66]. The study concluded that uncertainty in the temperature data was by far the most significant source of uncertainty. That study used observed data from a local weather station, widely considered the most reliable type of climate data available. Investments over the last 10 years in improved national climate data have demonstrated their value for use in national malaria decision-making [39]. Global climate-driven malaria models are developed using global climate datasets. Such datasets are widely available but are based on sparse meteorological observations, particularly in Africa [67]. These models are, therefore, only as reliable as the data that went into them. They must be interpreted with caution, especially when they are used to project into the future.

When making predictions using a climate-driven disease model, the climate data are themselves the output of a model (e.g. a weather or seasonal forecast, or a climate change projection), and thus add further to the overall uncertainty. As a general rule, the further into the future one predicts, the less specific one can be about what the climate will look like on local scales and at specific times. Short-term weather forecasts can be informative at very high resolutions, while seasonal climate forecasts are provided as area averages over areas ranging from sub-national to subcontinental scales. Consequently, climate-driven malaria predictions on longer timescales can provide only broad indications of potential transmission changes, rather than precise local information for direct use in malaria interventions.

To generate scenarios of how climate change could affect malaria, global transmission models are driven with the output of global climate models run several decades into the future [2]. There are some features of climate change projections that are critical to the interpretation of these scenarios:

- First, any future climate change scenario is contingent on the projected trajectory of greenhouse gas and aerosol emissions which drive climate change (and these trajectories themselves rest on a host of assumptions).
- Second, while climate models have been shown to reproduce some important aspects of the observed climate system, mostly on larger scales, they have many documented failings [8, 68–72]. There is thus considerable uncertainty about how the climate system will respond to the external forcing introduced by greenhouse gas emissions. Projections among models can differ dramatically, especially on regional or national scales, with some even predicting opposite changes in rainfall in many parts of the world [40].
- Third (and often overlooked), the timing of natural climate variations on interannual-to-decadal timescales cannot be predicted by climate change projections. Projections thus cannot be used to predict whether a particular year or decade is going to be wet or dry, but only to examine the general trend. In any given season, year or decade, the climate we experience can differ markedly from the projected trend, even if the trend turns out to be correct (Fig. 2). A projection that East African rainfall is likely to increase by the end of the twenty-first century says nothing about the trajectory between now and that future date. Even if average conditions become wetter, as predicted, fluctuations in climate will still result in periods of drought, flooding, and everything in between. The next 10–30 years (often called near-term climate change) is of great interest for planning, but poses exceptional challenges from a climate prediction standpoint. On this timeframe, interannual-to-decadal variability is crucial, but climate projections cannot be used to predict the timing of these fluctuations. In most places, rainfall trends are less significant on this timeframe, so the precipitation projections are of limited value. Warming trends, accompanied by intensifying heat extremes, are more prominent on these timescales, are expected to continue and should be factored in to long-term research and development activities. However, the precise location and timing of important changes (e.g. reaching key temperature transmission thresholds in new regions, or possible changes in seasonality) are highly uncertain.
- Fourth, climate models deliver data at a spatial resolution of approximately 50–100km^2^. Clearly, rainfall, temperature and other climate conditions change on much smaller spatial scales, particularly in mountainous areas which are an important boundary transmission zone. Model outputs, therefore, cannot be used directly to inform practical decision-making at national and local scales.
- Finally, climate model errors and uncertainty about current and future greenhouse gas and aerosol concentrations are addressed using ensembles: multiple simulations run using different initial conditions, emissions trajectories and climate models in an attempt to sample the full range of possible future climates. Studies projecting the effects of climate change on malaria must therefore examine the full spread of these ensembles. However, even the full ensemble of simulations is not designed to represent the true range of possible futures; that would be an impossible task. Thus, while it is hoped that the true future climate will lie somewhere within the range of projections, there is certainly no guarantee.

While model and scenario uncertainty are sometimes recognized in climate-driven disease modelling studies, natural variability is routinely handled incorrectly. It is common for such studies to compute trends in climate by choosing a single year, or 5–10 year period, which could result in serious over- or underestimation of the real trend (Fig. 2a).

### Implications for designing a malaria eradication strategy

In highlighting the scope and limitations of climate-driven projections of future malaria, the objective here is not to paralyse policy makers by pointing out the futility of guessing what the future holds. Nor do the authors want to minimize the substantial progress that has been made in the field of climate-driven disease prediction [73–76] or the utility of these models when used in the right way. Rather, the intention is to motivate a shift in the way climate is accounted for in the eradication strategy of the WHO. One simply cannot know whether climate models have captured the true range of plausible future climates, nor the impacts that these different climate conditions could have on malaria risk, particularly when the indirect effects of climate on socioeconomic systems are considered. Even if long-term climate trends transpire exactly as projected, climate shocks and variability are guaranteed to occur (Fig. 2), and will impact upon both transmission risk and control efforts (Fig. 1).

Climate-driven disease projections have their place within the long-term planning process, as tools for scenario planning and stress-testing existing systems and adaptation strategies [8]. They also have a role in shifting attitudes and motivating climate and health policy [9]. However, they are not suitable for planning specific practical measures and they must be used with some important rules of thumb in mind:

- Projections should only be used on continental to global scales, never to plan local responses or draw conclusions about future disease incidence at local scales
- Projections can only be used to infer broad climate conditions over periods of at least 30 years, never in a particular year or decade
- The possibility of outcomes occurring outside the range of projected futures should be accounted for explicitly in adaptation planning
- Projections should be regarded as plausible future scenarios, not predictions
- Projections should never be used without consulting a climate scientist to perform a thorough evaluation of the climate models on the timescale and for the locations of interest

Based on projected trends from one climate model, the Lancet commission on malaria eradication and the SAG_ME_ both conclude that climate can be dismissed as a factor that could hinder eradication. This simplistic guidance encourages an inflexible eradication programme that is optimized to one particular modelled scenario and which could, therefore, fail if that scenario turns out to be incorrect. Moreover, such a strategy could easily be derailed by climate shocks and variability, which have not been considered. It is critical that the malaria control programme that the WHO pursues is robust to a wide range of possible outcomes for the climate, and indeed for other uncertain drivers of malaria risk such as land use changes [77] or migration patterns. The SAGme categorizes these drivers of malaria risk (including climate) as *global trends* rather than *threats to eradication*. Climate would be more appropriately classified as a threat to eradication, both because of uncertainty in future climate change and the role of climate shocks and variability as drivers of transmission and as key factors in control efforts. In fact, climate is relevant to all four priority areas for the SAGme: research and development for new tools; access to health services; surveillance and response; and subnational, national and regional strategies. It should be mainstreamed within the programmes for each of these priority areas to ensure that the threats that climate poses to malaria control, as well as the opportunities, are managed appropriately.

## Climate-proofing a malaria eradication strategy

### Using climate information for decision making

Climate provides a number of unique opportunities when compared with other factors that affect malaria transmission such as co-infection or human behaviour. To begin with, climate varies predictably by location according to defined processes. Climate is also routinely measured, modelled and predicted at multiple space and timescales using structured methods. Despite many known weaknesses, this highly structured mass of data, stored in national and global repositories, provides hourly, daily, weekly and monthly historical information for most regions of the planet and forms the basis for predictions of the future climate. This invaluable resource is largely underutilized by the public health community. Climate information, which may include historical and/or real-time data as well as forecasts, has the potential to inform a wide range of health decisions [78] through an improved understanding of: mechanisms of disease transmission; spatial risk (by identifying populations at risk to better-target interventions); routine seasonal risks (to inform the timing of routine interventions like indoor residual spraying); sub-seasonal to interannual changes in risk (through early warnings of potential changes in epidemic risk based on climate monitoring and forecasts (Box 1)); trends in risk (to identify and plan for long-term drivers of disease occurrence); and impact assessments (to improve interventions by removing climate as a confounding factor in evaluations).

Different types of climate information are needed to inform the various components of malaria control and prevention programmes. Realizing the opportunities afforded by climate thus rests on providing information at the appropriate timescale and spatial resolution to correspond to particular activities (Table 1). For example, malaria control practitioners may be interested in ensuring that the malaria control strategy is resilient to climate change but, as they work within a traditional political cycle and with annual budgets, long term climate scenarios are unlikely to be relevant to their immediate decisions. Conversely, national and international policy makers concerned with the development of long-term strategies, medical and vector counter measures and research and development may need to consider the impact of warming on the strategies and products they develop. Failure to target climate services at the right space and timescales will either result in no action at all, as practitioners struggle to identify the appropriate response, or in misinformed decision-making that could increase vulnerability to climate variability and change.

**Table 1:** Time horizons for decision-making in the health sector [82]

Investment 2–5 decades	Carbon emissions mitigation strategies
	Malaria eradication strategies
	Major infrastructure investment
	Workforce development
Strategic planning 6–20 years	Research and development of medical countermeasures (e.g., drugs, vaccines) and vector control tools (e.g., new insecticides)
	Improving the nutritional content of crops
	Health facility investments
	Curriculum development
Policy cycles 2–5 years	4- to 5-year political cycle
	Health service re-organization
	2- to 5-year research grant cycle
Planning cycles < 2yrs	Annual planning and commissioning cycle
	Demand for visible ‘quick wins’ from funders
Seasonal preparedness and response < 4 months	Seasonal planning cycle
	Epidemic/disaster preparedness and response
Weekly facility management < 1 week	Weather disaster preparedness and response
	Patient scheduling for non-urgent cases

**Fig. 3:**
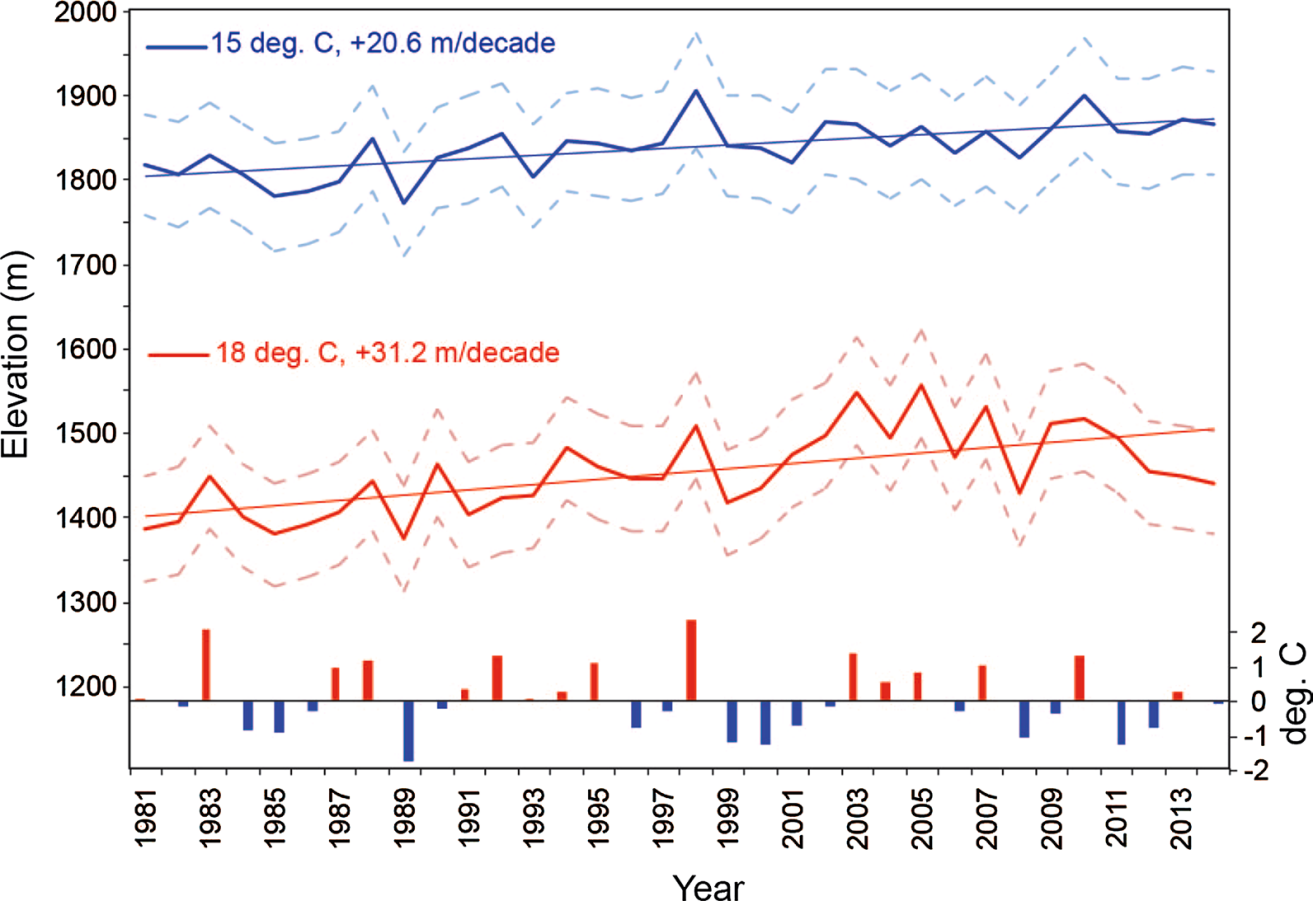
Changes in the elevation threshold for malaria over time in the Ethiopian Highlands. Solid lines: mean elevation above mean sea level (m) where the 10-day average minimum temperature in a year never exceeds 18 °C (red) and 15 °C (blue). Dashed lines indicate the uncertainty in mean elevation. Coloured bars show anomalous values (°C) of the October–December “Niño 3.4” sea surface temperature index in the Pacific Ocean. Trend lines and associated slopes are also shown [42]

Box 1: Practical entry points for managing long-term climate trends Although climate shocks and variability result in much larger changes in rainfall and temperature than long-term trends in most locations, trends are also important drivers of malaria risk. An example from the Ethiopian highlands illustrates well how long-term trends can be managed within an eradication programme, given the challenges of accurately predicting them. The decrease in temperature with elevation in mountainous areas provides a natural barrier to malaria outbreaks as, above a certain elevation, night-time temperatures drop below the minimum temperature limit for parasite survival. As the climate has warmed, highland areas have seen a corresponding increase in the altitude at which malaria transmission is possible, exposing non-immune populations in new highland areas to risk of the disease [42]. The increase in this threshold elevation has not been steady, but varies from year to year, in part driven by the occurrence of El Niño and La Niña events in the Pacific Ocean, which cause local temperatures to fluctuate. Climate change is thus not acting alone to influence local malaria risk; rather, long-term temperature trends are altering the extent to which normal climate variability impacts on disease risk. The entry point to addressing this worsening problem is not through uncertain projections of temperature trends decades from now, but through seasonal early warning systems based on observed or forecast seasonal temperatures. Seasonal early warning systems can provide sufficient notice to scale up control efforts in highland regions if needed, as long as protocols are already in place to ensure that resources can be mobilized efficiently (Fig. 3).

### Practical recommendations

#### Begin with managing short-term climate risks to control and elimination activities

Complete eradication is a long-term goal which, in practice, will be achieved through effective control and elimination programmes. Most practical planning decisions in these programmes have time horizons from days to a few years (Table 1) and are sensitive to the weather and to seasonal and interannual climate shocks and variability. These risks are best managed by making use of historical data to understand the role of past climate events in control efforts, combined with up-to-date monitoring of climate conditions. If additional time for early action is needed, climate forecasts can also be used, provided they are evaluated and prove skillful.

#### Incorporate climate into monitoring and evaluation of malaria control efforts

Knowledge about the role climate may have played in specific malaria outbreaks will help to understand when and how malaria control efforts are effective and better target these strategies to achieve maximum public health benefit.

#### Invest in monitoring and surveillance systems for climate and malaria

To enable this knowledge base to be built, investments are needed in both malaria surveillance and in climate observing systems. These improved surveillance systems are needed both within current malaria endemic zones where the disease is increasingly controlled as well as on the margins of transmission to identify areas of emerging risk. Climate observations are particularly lacking in large areas of Africa. The ENACTS (Enhancing National Climate Services) initiative mitigates this problem by combining quality-controlled data from national observing networks with rainfall estimates from satellites, incorporating elevation and reanalysis temperature products. The result is a high-resolution gridded dataset within national repositories, incorporating the best available climate data from different sources. Derived products can then be made available for use in decision-making [67, 79].

#### Iteratively review and update malaria eradication strategies

Flexible plans can be iteratively updated to respond to recent observations of climate changes to date, such as changes in seasonality or trends that alter the climate suitability for transmission in boundary zones. In active transmission zones, monitoring and/or forecasts may indicate higher climatic suitability for malaria in the coming months, or conversely, favourable conditions that provide an opportunity to push for elimination. In regions where decadal variability is strong, strategies should also make use of long-term monitoring data to respond to shifting decadal cycles with appropriate disease control strategies.

#### Long-term investments

In rare cases where non-flexible planning and investment are needed on very long-term time frames (Table 1), these investments should be guided by several lines of evidence: sensitivity studies that stress-test the tolerance of (for example) vaccines or insecticides to a range of potential changes in climate to highlight particular vulnerabilities; analyses of observed changes in climate to date that can identify where critical climate thresholds are being approached or crossed; where changes in specific climate events are a concern, analyses of the large-scale climate drivers of these events and the plausibility of reaching important thresholds, based on model projections and an assessment of whether the underlying physics supports them [8].

#### Build capacity and partnerships

Fostering partnerships between climate and health communities will facilitate the development and uptake of high-quality, fit-for-purpose datasets for use in health-policy decision making. Capacity building on the use of climate information for malaria decision making is needed among malaria policy makers and practitioners, while climate service providers require training on the types of climate information that can best inform malaria control programmes and how this information should be communicated to assist decision making. Building alliances between malaria programmes, ministries of health and relevant international environmental and development partners can also be a means of securing access to adaptation funds to manage climate-related risks to the success of malaria programmes [80].

### Research priorities

The following research priorities are proposed to facilitate the recommended programmatic activities. Progress on this research agenda will depend on sustained investments in surveillance systems for climate and malaria as described above (activity 3). Related programmatic recommendations from the above list are indicated in parentheses for each research priority:

- Diagnosing the role of climate *vs.* non-climate factors in the success and failure of previous malaria interventions in order to better target future efforts and justify funding for specific malaria control strategies [activities 1 & 2].
- Regular evaluations of the malaria suitability of recent climate conditions in marginal and endemic transmission zones, in conjunction with other lines of evidence such as case rates, to identify areas of emerging risk and to course-correct eradication plans as needed (e.g. every 5 years) [activities 1, 2 & 4].
- Testing the sensitivity of malaria control and elimination strategies to plausible, hypothetical, changes in future climate (including the potential expansion of malaria zones). Stress tests can identify key vulnerabilities within programmes that could be reduced, both to improve current performance and to make future plans more robust to climate change uncertainty, without relying on projections [activities 4 & 5].
- If important long-term decisions must be made that are highly sensitive to future climate conditions, analyses should be conducted to assess appropriate levels of confidence in the projections on the timescales and in the locations relevant for the decisions in question. Such assessments require bespoke analyses to diagnose where the models perform well, where they fail and why, supported by an understanding of the scientific plausibility of the projections. They cannot be shortcut through a one-size-fits-all approach [8, 81] [activity 5].
- Investigating methods to depict and communicate plausible future climate scenarios in ways that facilitate robust decision making within disease programming [activities 4, 5 and 6].
- Identifying case studies that illustrate effective and less effective uses of climate information for malaria control, with particular consideration to how successful examples might be scaled up [activities 1, 2, 4, 5 & 6].

## Data Availability

The datasets analysed during the current study are available in the IRI Data Library repository at http://iridl.ldeo.columbia.edu/maproom/Global/Time_Scales/index.html. Other materials used to provide evidence for the study were taken from published sources which are appropriately referenced herein.

## Abbreviations

SAG_ME_: Strategic Advisory Group on Malaria Eradication
WHO: World Health Organization
IRS: Indoor Residual Spraying
