# Correction to: Climate-proofing a malaria eradication strategy

**DOI:** 10.1186/s12936-021-03747-6

**Published:** 2021-05-10

**Authors:** Hannah Nissan, Israel Ukawuba, Madeleine Thomson

**Affiliations:** 1 Grantham Research Institute for Climate Change and the Environment, London School of Economics and Political Science, London, UK; 2 International Research Institute for Climate and Society, Columbia University, Palisades, NY USA; 3 Mailman School for Public Health, Columbia University, New York, NY USA; 4 Columbia University, New York, NY USA

## Correction to: Malar J (2021) 20: 190 10.1186/s12936-021-03718-x

Following publication of the original article [1], it was brought to our attention that the entries under the section ‘Practical recommendations' had not been numbered, when they should have been numbered as follows:

1. Begin with managing short-term climate risks to control and elimination activities
2. Incorporate climate into monitoring and evaluation of malaria control efforts
3. Invest in monitoring and surveillance systems for climate and malaria
4. Iteratively review and update malaria eradication strategies
5. Long-term investments
6. Build capacity and partnerships

Furthermore, the Box had been incorrectly formatted in the PDF version, with the last sentence placed outside the box.

The original article has since been corrected.

The publisher apologizes for any inconvenience caused.
